# Pharmacokinetics of indacaterol, glycopyrronium and mometasone furoate administered as an inhaled fixed-dose combination in Japanese and Caucasian healthy subjects

**DOI:** 10.1186/s12890-020-01382-6

**Published:** 2021-01-07

**Authors:** Satoru Inoue, Soniya Vaidya, Hanns-Christian Tillmann, Yohei Sakita, Surendra Machineni, Olivier Heudi, Kenichi Furihata

**Affiliations:** 1 Novartis Pharma KK, Tokyo, Japan; 2 Novartis Institutes for BioMedical Research, Cambridge, USA; 3 Novartis Institutes for BioMedical Research, Basel, Switzerland; 4 Novartis Healthcare Pvt. Ltd., Hyderabad, India; 5 P-One Clinic, Keikokai Medical Corporation, Tokyo, Japan

**Keywords:** Indacaterol/glycopyrronium/mometasone furoate, Pharmacokinetics, Japanese–Caucasian, Asthma

## Abstract

**Background:**

A once-daily (o.d.) fixed-dose combination of indacaterol acetate (IND), glycopyrronium bromide (GLY), and mometasone furoate (MF) delivered via the Breezhaler^®^ device (IND/GLY/MF) is being developed for treatment of asthma. This study compared steady-state pharmacokinetics of IND, GLY and MF between Japanese and Caucasian male subjects after multiple inhalations of IND/GLY/MF o.d.

**Methods:**

This was a single-center, open-label, 2-treatment crossover study with a 21-day washout period. Japanese and Caucasian subjects received IND/GLY/MF 150/50/80 μg (inhaled corticosteroid [ICS] medium-dose) or 150/50/160 μg o.d. (ICS high-dose) for 14 days in each period. Pharmacokinetics were characterized up to 24 h post-dose on Days 1 and 14.

**Results:**

In total, 16 Japanese (median age 31 years [range 20–40 years], mean weight 68.3 kg) and 17 Caucasian subjects (median age 27 years [range 21–43 years], mean weight 75.0 kg) were randomized. Geometric mean ratios (Japanese/Caucasian) [90% confidence interval (CI)] for C_max_ for IND, GLY and MF at the high ICS dose on Day 14 were 1.31 [1.13, 1.51] 1.38 [1.13, 1.69] and 1.07 [0.969, 1.18], respectively. Geometric mean ratios (Japanese/Caucasian) [90% CI] for AUC_0–24h_ on Day 14 for IND, GLY and MF at the high ICS dose were 1.17 [1.01, 1.35], 1.05 [0.920, 1.20] and 1.15 [1.05, 1.27] respectively. Similar trends were noted for all components for the medium ICS dose treatment. IND/GLY/MF was safe and well tolerated; no AEs suspected to be study drug-related were observed.

**Conclusion:**

Pharmacokinetics of IND, GLY and MF (high and medium dose) when delivered as a fixed-dose combination were comparable between Japanese and Caucasian subjects. The IND/GLY/MF combination at the administrated doses was safe and well tolerated in both ethnic groups.

**Trial registration:**

Japan Registry of Clinical Trial: jRCT2031200227, retrospectively registered on 04, December, 2020.

## Background

Asthma is a chronic inflammatory disorder associated with airway hyper-responsiveness, airway inflammation, and airway structural remodeling that leads to recurrent episodes of wheezing, breathlessness, chest tightness, and coughing, particularly at night or in the early morning [1]. As of 2017, 358 million individuals suffer from asthma worldwide, including an estimated 30 million people in Europe and 17.7 million people in the US [2]. In Japan, the Ministry of Health, Labor, and Welfare reported approximately 1,177,000 (515,000 men and 662,000 women) from asthma sufferers in 2014 [3]. The proportion of adult Japanese asthma patients with severe asthma and receiving step 4 treatment as per Japanese guidelines or step 5 as per GINA guideline was 10.6% [3]. Asthma patients who are uncontrolled on low-dose inhaled corticosteroid (ICS)/long-acting β agonist (LABA), despite good adherence and correct technique, may benefit from increasing the maintenance dose to medium-dose ICS-LABA or use of add-on long-acting muscarinic antagonist (LAMA) or add-on leukotriene receptor antagonists (LTRA) or high-dose ICS/LABA (Step 4) [4].

Non-adherence to medication is a major cause of poor control of asthma and may be related to several factors including difficulty using inhalers properly, complicated regimens (e.g. multiple times per day, multiple different inhalers) and misunderstanding of the role of controller medications [4]. Improvements in medication adherence could lead to significant improvements in asthma outcomes [5]. Therefore, the once daily combination of LABA/LAMA/ICS in a single inhaler can be an effective treatment option for patients with persistent asthma, while improving patient adherence to asthma therapy [6, 7]. Indacaterol acetate (IND), glycopyrronium bromide (GLY), and mometasone furoate (MF) delivered via the Breezhaler^®^ device (IND/GLY/MF) is a fixed-dose combination (FDC) of IND, a LABA, GLY, a LAMA, and MF, an ICS, in development for o.d.-maintenance treatment of asthma GINA (Step ≥ 4). Each of these three mono-components, IND [8–10], GLY [11, 12] and MF [13, 14] have previously been approved as individual drugs for either chronic obstructive pulmonary disease (COPD) or asthma. Ethnic factors are known to contribute to differences in pharmacokinetics (PK) and pharmacodynamics of a number of drugs, thereby, resulting in variations in the treatment response [15–17]. Studies have reported no differences in the systemic exposure of IND and GLY between Japanese and Caucasian subjects upon administration via the Breezhaler^®^ device [18, 19]. However, for MF delivered with the Twisthaler^®^ inhalation device, the maximum plasma concentration (C_max_) and area under the curve (AUC) values were about two to threefold higher for Japanese patients with asthma compared with Caucasian patients with asthma [20].

The present Phase I study aims to investigate a potential impact of ethnic factors on the PK of orally inhaled IND/GLY/MF 150/50/80 μg (medium-dose ICS) or 150/50/160 μg (high-dose ICS) o.d., delivered via the Breezhaler^®^ device in healthy Japanese and Caucasian subjects.

## Methods

**Fig. 1:**
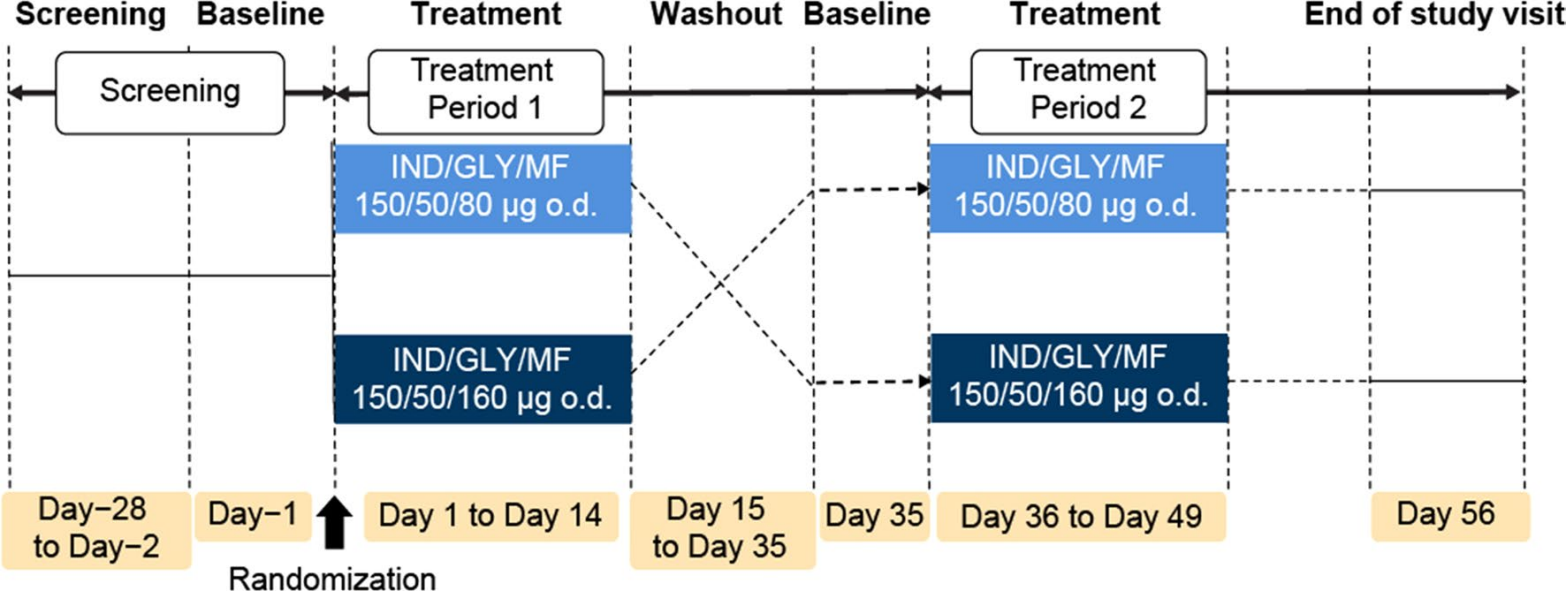
Study design. IND/GLY/MF is a combination of indacaterol acetate 150 μg, glycopyrronium bromide 50 μg and mometasone furoate 80 μg. (medium-dose ICS) or 160 μg (high-dose ICS) delivered o.d. via the Breezhaler^®^. *GLY* glycopyrronium bromide, *IND* indacaterol acetate, *MF* mometasone furoate, *o.d.* once daily

This was a single-center, open-label, two-treatment crossover study with a 21-day washout period. Eligible Japanese and Caucasian subjects were randomized to one of two treatment sequences and received IND/GLY/MF 150/50/80 μg (medium-dose ICS) [capsules for inhalation, Novartis Pharma AG, Batch number: J17012) or 150/50/160 μg (high-dose ICS) [capsules for inhalation, Novartis Pharma AG, Batch number: J17013) o.d., as a single oral inhalation, delivered via the Breezhaler^®^ device for 14 days in each treatment period (Fig. 1). All subjects were trained in the use of the Breezhaler^®^ device at the screening visit and the baseline visits of both treatment periods. All treatments were followed by post-inhalation mouth rinsing using two rinses of 30 mL water. Water used for mouth rinsing was to be spat out. Subjects were instructed to not swallow the water. The particle size for MF in both treatments was similar, while the in vitro fine particle mass (FPM) increased in a dose proportional manner between the two MF doses (80 μg and 160 μg). The FPM is a measure of the quantity of smaller (≤ 5 µm) particles delivered from the inhaler that generally deposit in the airway and can be considered as an indicator of lung deposition [21]. The study consisted of a screening visit, two treatment sequences, each with a baseline and treatment period, a washout period and an end of study (EOS) visit. Eligible subjects were randomized to one of two treatment sequences and received IND/GLY/MF (high- or medium-dose, Fig. 1) for 14 days in treatment period 1 after an initial 2- to 28-day screening period and baseline evaluation (Day-1).

Following this, there was a 21-day washout period between the last treatment of treatment period 1 and the first treatment in treatment period 2, prior to treatment administration in period 2. The subjects remained at the study center during each treatment period. The study completion visit was performed on Day 21 of period 2, at which time drug safety was evaluated. At the baseline visit of both treatment periods, all subjects were trained in the appropriate use of the Breezhaler^®^ device. PK was evaluated up to 24 h post-dose on Days 1 and 14 of each period. On Day 1, 14 of each treatment period, all subjects fasted (i.e. no food and liquid except water) for at least 10 h prior to administration of study treatment and continued to fast for at least 4 h thereafter. For the other dosing days (Day 2 to 13 of each treatment period), study medication was administered before breakfast and no overnight fast was required. On Day 1 and 14 of each treatment period, no fluids were permitted, apart from the fluid given for mouth rinsing following the inhalations, for 1 h prior to and 1 h after study drug administration. Otherwise, subjects had a fluid intake of approximately 200 mL every 4 h during waking hours in addition to fluid taken with meals. There were no fluid restrictions on any other dosing days (Day 2–13 of each treatment period).

The study was conducted in accordance with the principles of the Declaration of Helsinki, the International Conference on Harmonization Guideline for Good Clinical Practice, and the Japanese Standards for the Implementation of Clinical Trials on Pharmaceutical Product. The study protocol was reviewed and approved by the Institutional Review Board of P-one clinic.

## Study objectives

The primary objective was to compare the steady-state PK of IND/GLY/MF between Japanese and Caucasian healthy male subjects after multiple o.d. oral inhalation of IND/GLY/MF medium and high dose. The key secondary objective was to compare the PK of IND/GLY/MF between Japanese and Caucasian healthy male subjects after single-dose oral inhalation of IND/GLY/MF medium or high dose, via Breezhaler^®^ on Day 1. The exploratory objective was to compare the PK of MF between high-dose ICS (MF dose: 160 μg) and medium-dose ICS (MF dose: 80 μg) on Day 1 and Day 14, respectively. MF Twisthaler and MF Breezhaler^®^ are different in regards to both the inhalation device and the formulations they deliver. The medium and high MF doses (80 μg and 160 μg) as part of the IND/GLY/MF combination delivered via the Breezhaler^®^ device are comparable to the 400 μg and 800 µg doses of MF delivered by the Twisthaler device, respectively. The high MF dose (320 μg) as part of the IND/MF combination delivered via the Breezhaler^®^ device is comparable to the 800 μg dose of MF delivered by the Twisthaler device [22]. The high MF dose (160 μg) as part of the IND/GLY/MF combination is comparable to the high MF dose (320 μg) as part of the IND/MF combination in terms of systemic exposure [23].

## Study population

A total of 32 subjects (16 Japanese and 16 Caucasian) were planned to be randomized in this study. Each Caucasian subject was to be matched pair wise to his Japanese counterpart by age (± 10 years) and weight (± 20% [kg]). All subjects provided written informed consent prior to any assessments being performed. As per the key inclusion criteria, participants were healthy male subjects, aged 20–45 (inclusive) years of age, a BMI of 18–30 kg/m^2^ and in good health as determined by past medical history, physical examination, vital signs, electrocardiogram, and laboratory tests at screening and baseline of treatment period 1. Japanese subjects were to be of first-generation Japanese origin (Japanese subjects of first generation are defined as subjects living in Japan and having both parents and grandparents of Japanese descent); Caucasian subjects of first-generation Caucasian origin (Caucasian subjects of first generation are defined as subjects having both parents and grandparents of Caucasian descent). Key exclusion criteria were: (1) use of other investigational drugs at screening, or within five half-lives of enrollment, or within 4 months, whichever was longer; (2) a history of clinically significant ECG abnormalities; (3) significant illness which had not resolved within 2 weeks prior to initial dosing; (4) history of acute or chronic bronchospastic disease (including asthma and COPD, treated or not treated); (5) any surgical or medical condition which might significantly alter the absorption, distribution, metabolism, or excretion of drugs, or which could jeopardize the subject in case of participation in the study; (6) inability to use the Breezhaler^®^ device at screening.

## Pharmacokinetic analysis

For the PK assessments of IND, GLY and MF, blood samples were collected pre-dose, and at 5 min (IND and GLY only), 15 min, 30 min, 1 h, 2 h, 3 h, 6 h, 12 h, and 24 h after dosing on Days 1 and 14. Assessment windows were: 30 min from study drug administration for pre-dose samples on Day 1, − 10 min from study drug administration for pre-dose samples on Day 14, ± 3 min for 5 min, 15 min post-dose samples, ± 5 min for 30 min, 1 h, 2 h and 3 h post-dose samples, ± 10 min for 6 h, 12 h and 24 h post-dose samples.

All blood samples were taken by either direct venipuncture or an indwelling catheter inserted in a forearm vein. At specified time points, 4 mL blood was collected in lithium heparin tubes for determination of IND and GLY, and 6 mL blood was collected in K2-EDTA tubes for determination of MF. The plasma samples of each compound were assayed using three separate validated liquid chromatography tandem mass spectrometry methods. Briefly, for IND determination, 0.2 mL plasma was processed using solid phase extraction and then separated on an ACQUITY UPLC BEH C_18_ column using acidified water and acetonitrile mobile phase at a flow rate of 0.750 mL/min within 3.30 min. The lower limit of quantification (LLOQ) of IND was 5.00 pg/mL. For GLY determination, sample processing was performed by means of solid phase extraction using a volume of 0.2 mL plasma. Separation was obtained within 2.5 min on an ACQUITY UPLC BEH C_18_ analytical column at a flow rate of 0.750 mL/min with binary mobile phases made of water and acetonitrile both acidified 0.1% formic acid. The LLOQ of the method was 1.00 pg/mL.

For the MF determination, the method consisted of liquid–liquid extraction using 0.8 mL of plasma, and the separation of the extracted sample was achieved on an ACQUITY UPLC BEH C_18_ column at a flow rate of 1.00 mL/min within 3.51 min, using a binary mobile phase made of 0.05% ammonia in water and acetonitrile. The LLOQ of the method was 0.250 pg/mL. Additional analytical parameters and performances of the three methods used for the determination of the three compounds are summarized in Additional file 1: Table 1. Concentrations below the LLOQ were treated as zero in summary statistics of concentration data as well as PK parameter calculations. PK parameters (AUC_0–24 h_, C_max_ and T_max_) were determined by non-compartmental analysis using WinNonlin Phoenix (version 6.4; Certara, Princeton, NJ, USA).

## Safety

Safety was monitored in terms of adverse events (AEs) and serious adverse events (SAEs). There was regular monitoring of hematology, blood chemistry, urinalysis, and ECGs, and regular assessments of vital signs, physical condition, and bodyweight.

## Statistical analysis

All subjects who received at least one dose of study medication were included in the safety analysis set. The PK analysis set included all subjects with at least one available valid PK concentration measurement, who received any study drug and had no protocol deviations that were expected to impact the PK analysis. Log-transformed primary PK parameters (AUC_0–24 h_ and C_max_) for IND, GLY and MF on Day 1 and Day 14 were analyzed by day using a linear mixed-effects model. The model included ethnic group, sequence, period, treatment and the interaction between ethnic group and treatment as fixed effects. Subject and matched pairs were considered as random effects. All matched pairs with evaluable PK parameters for at least one subject were included in the analysis. The above analysis was also repeated by including baseline age and bodyweight as covariates into the model.

## Results

### Study participants

A total of 33 subjects (16 Japanese and 17 Caucasian subjects) were randomized to one of the two IND/GLY/MF treatment sequences. Twenty nine out of 33 subjects (14 Japanese and 15 Caucasian subjects) completed the study while four subjects (2 Japanese and 2 Caucasian subjects) discontinued the study. Three subjects (one Japanese and two Caucasian subjects) receiving IND/GLY/MF 150/50/80 μg discontinued the study due to the withdrawal of consent by subject in treatment period 1. One Japanese subject receiving IND/GLY/MF 150/50/80 μg was discontinued from the study by the Investigator due to AEs of gastroenteritis and headache, in treatment period 2.

All the subjects were men; the baseline characteristics are presented in Table 1. The study population of healthy Japanese and Caucasian subjects was consistent with the key inclusion and exclusion criteria as outlined under study population in the Methods section. The median age of Japanese subjects was 31 years and median age of Caucasian subjects was 27 years. Mean bodyweight was higher in Caucasian subjects than in Japanese subjects.

**Table 1:** Demographic characteristics

	Japanese N = 16	Caucasian N = 17
Age (years), median	31.0	27.0
Range	20–40	21–43
Weight (kg), mean ± SD	68.25 ± 6.10	75.01 ± 7.99
Height (cm), mean ± SD	173.3 ± 4.1	178.7 ± 4.50
Body mass index (kg/m^2^), mean ± SD	22.69 ± 2.07	23.44 ± 2.32

### Pharmacokinetics

#### Indacaterol

**Fig. 2:**
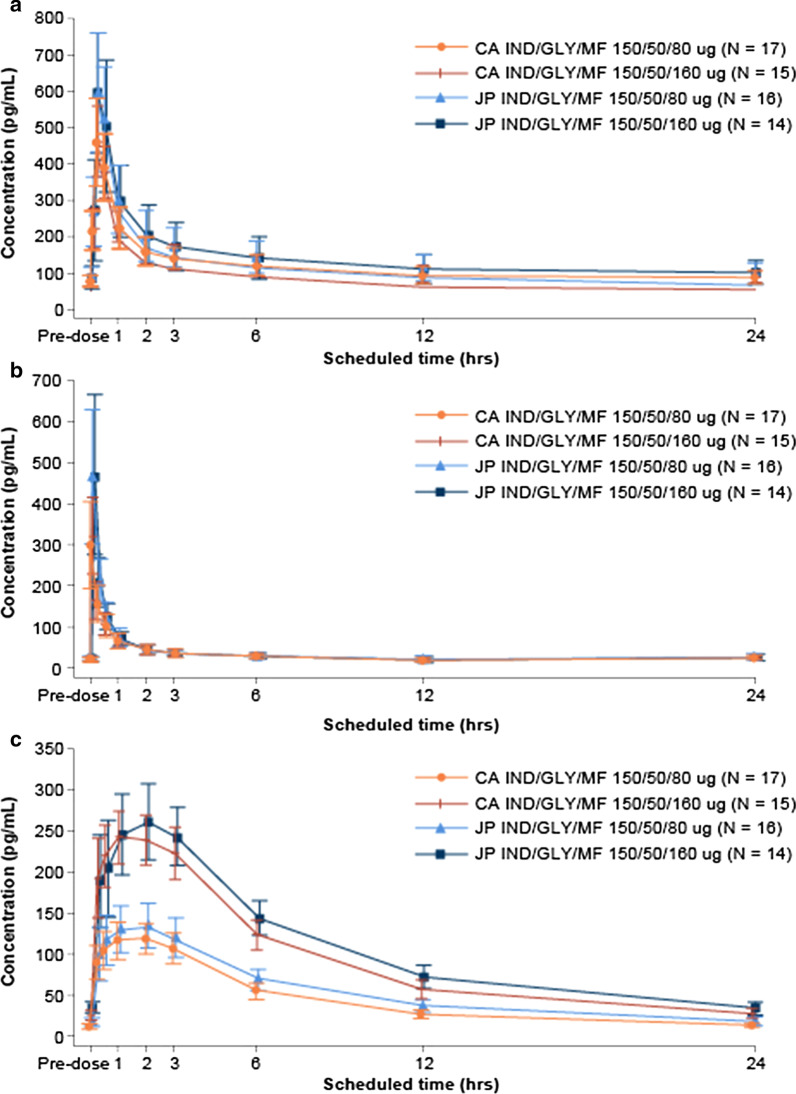
Mean concentration–time profile of **a** indacaterol, **b** glycopyrronium and **c** mometasone furoate on Day 14. Data presented as mean ± SD. IND/GLY/MF is a combination of indacaterol acetate 150 μg, glycopyrronium bromide 50 μg and mometasone furoate 80 μg (medium-dose ICS) or 160 μg (high-dose ICS) delivered o.d. via the Breezhaler^®^. *CA* Caucasians, *GLY* glycopyrronium bromide, *IND* indacaterol acetate, *JP* Japanese, *MF* mometasone furoate, *o.d.* once daily

Overlapping mean plasma concentration–time profiles of IND were observed between treatment groups (IND/GLY/MF medium and high-dose) in both Japanese and Caucasian subjects. The mean plasma concentrations were slightly higher in Japanese subjects than in Caucasian subjects (Fig. 2a). Following single and multiple doses of IND/GLY/MF (either medium or high dose), the median T_max_ of IND was 0.25 h, on both Day 1 and Day 14. The accumulation ratios (R_acc_ for AUC_0–24 h_ and C_max_) after o.d. dosing for 14 days were 3.09–3.32 and 1.57–1.76, respectively in Japanese subjects, and were 3.30–3.31 and 1.74–1.75 in Caucasian subjects (Table 2). Following multiple doses of IND/GLY/MF, the geometric mean ratios (Japanese/Caucasian) for C_max_ on Day 14 in IND/GLY/MF high- and medium-dose treatment groups were 1.31 (90% CI 1.13–1.51) and 1.30 (90% CI 1.13–1.49), respectively. The geometric mean AUC_0–24 h_ on Day 14 in IND/GLY/MF high- and medium-dose treatment groups were 1.17 (90% CI 1.01–1.35) and 1.21 (90% CI 1.05–1.40), respectively. The geometric mean C_max_ and AUC_0–24 h_ values were slightly higher up to 1.31-fold for C_max_ and 1.21-fold for AUC_0–24 h_ in Japanese subjects than in Caucasians. Similar trends were observed for single doses of IND/GLY/MF medium and high dose on Day 1 (Table 3). In the exploratory statistical analysis including age and bodyweight as covariates, the geometric mean ratios (90%CI) (Japanese/Caucasian) for C_max_ on Day 14 in IND/GLY/MF medium- and high-dose treatment groups for IND were 1.20 (1.04–1.40) and 1.22 (1.05–1.42), respectively. The geometric mean ratios (90% CI) (Japanese/Caucasian) for AUC_0–24 h_ on Day 14 in IND/GLY/MF medium- and high-dose groups were 1.17 (1.00–1.36) and 1.12 (0.96–1.31), respectively.

**Table 2:** Summary of PK parameters after single (Day 1) and multiple doses (Day 14) of IND/GLY/MF administered via oral inhalation in healthy subjects

		Japanese		Caucasian	
PK parameters (unit)	Profile day	IND/GLY/MF medium-dose N = 15/16	IND/GLY/MF high-dose N = 13/14	IND/GLY/MF medium-dose N = 17	IND/GLY/MF high-dose N = 15
Analyte: indacaterol					
C_max_ (pg/mL)	Day 1	388 (25.3)	338 (28.7)	275 (31.8)	282 (33.0)
	Day 14	595 (27.2)	593 (27.9)	460 (25.8)	461 (21.1)
AUC_0–24 h_ (h_×_pg/mL)	Day 1	1090 (25.0)	1010 (34.5)	862 (25.8)	897 (28.6)
	Day 14	3360 (30.3)	3330 (37.1)	2750 (23.0)	2800 (20.3)
T_max_ (h)	Day 1	0.250 (0.250–0.500)	0.250 (0.250–0.250)	0.250 (0.250–0.250)	0.250 (0.250–0.250)
	Day 14	0.250 (0.250–0.250)	0.250 (0.250–0.250)	0.250 (0.250–0.250)	0.250 (0.250–0.250)
R_acc_ (AUC)	Day 14	3.09 (17.8)	3.32 (24.2)	3.31 (25.2)	3.30 (27.8)
R_acc_ (C_max_)	Day 14	1.57 (20.7)	1.76 (22.1)	1.75 (25.8)	1.74 (27.1)
Analyte: glycopyrronium					
C_max_ (pg/mL)	Day 1	318 (53.5)	296 (47.6)	204 (46.2)	215 (48.9)
	Day 14	467 (35.1)	464 (42.7)	300 (34.7)	322 (28.9)
AUC_0–24 h_ (h_×_pg/mL)	Day 1	294 (25.3)	278 (31.5)	276 (35.2)	280 (33.0)
	Day 14	772 (21.0)	751 (17.5)	734 (25.3)	734 (20.7)
T_max_ (h)	Day 1	0.0833 (0.0833–0.0833)	0.0833 (0.0833–0.0833)	0.0833 (0.0833–0.0833)	0.0833 (0.0833–0.0833)
	Day 14	0.0833 (0.0833–0.0833)	0.0833 (0.0833–0.0833)	0.0833 (0.0833–0.0833)	0.0833 (0.0833–0.0833)
R_acc_ (AUC)	Day 14	2.74 (27.5)	2.86 (25.4)	2.84 (30.2)	2.83 (28.4)
R_acc_ (C_max_)	Day 14	1.69 (41.8)	1.80 (46.3)	1.68 (42.4)	1.90 (65.2)
Analyte: mometasone furoate					
C_max_ (pg/mL)	Day 1	105 (16.3)	197 (18.3)	98.6 (22.3)	195 (16.5)
	Day 14	141 (18.8)	268 (18.9)	123 (17.9)	252 (14.8)
AUC_0–24 h_ (h_×_pg/mL)	Day 1	936 (15.0)	1730 (16.8)	800 (19.9)	1620 (19.2)
	Day 14	1270 (15.8)	2540 (14.0)	1040 (16.2)	2220 (12.6)
T_max_ (h)	Day 1	2.00 (0.500–3.00)	1.00 (0.250–2.00)	1.00 (0.250–2.00)	2.00 (0.250–2.00)
	Day 14	2.00 (0.250–3.00)	2.00 (0.250–3.00)	1.00 (0.250–3.00)	1.00 (0.250–2.00)
R_acc_ (AUC)	Day 14	1.37 (14.2)	1.50 (20.1)	1.33 (17.1)	1.40 (12.8)
R_acc_ (C_max_)	Day 14	1.35 (15.3)	1.38 (18.6)	1.28 (19.8)	1.31 (17.5)

Statistics are arithmetic mean (CV%); for T_max_, statistics are Median (Min–Max). The accumulation ratio (R_acc_) parameters are derived as the ratio between the profile day shown and the Day 1 results. IND/GLY/MF medium-dose, IND/GLY/MF 150/50/80 μg o.d; IND/GLY/MF high-dose, IND/GLY/MF 150/50/160 μg o.d. AUC, area under curve; C_max_, maximum plasma concentration; IND/GLY/MF, indacaterol/glycopyrronium/mometasone; R_acc_, accumulation ratios; T_max_, The time to reach the maximum concentration after drug administration. N varies across treatments and ethnicities based on treatment discontinuations during the study as described under study participants. For two Japanese subjects (one profile for the high dose treatment and one profile for the medium dose treatment) for whom pre-dose indacaterol concentration on Day 1 was missing, Day 1 PK parameters were excluded from primary analysis

**Table 3:** Summary of the analysis of plasma PK parameters (Japanese vs Caucasian) on Day 1 and Day 14

		Day 1			Day 14		
		Adjusted geometric mean		Adjusted geometric mean ratio (90% CI) (Japanese/Caucasian)	Adjusted geometric mean		Adjusted geometric mean ratio (90% CI) (Japanese/Caucasian)
Treatment	PK parameter (unit)	Japanese	Caucasian		Japanese	Caucasian	
Analyte: indacaterol							
IND/GLY/MF high-dose	C_max_ (pg/mL)	327 (n = 13)	269 (n = 15)	1.22 (1.05, 1.41)	586 (n = 14)	447 (n = 15)	1.31 (1.13, 1.51)
	AUC_0–24 h_ (h × pg/mL)	989 (n = 13)	877 (n = 15)	1.13 (0.971, 1.31)	3190 (n = 14)	2730 (n = 15)	1.17 (1.01, 1.35)
IND/GLY/MF medium-dose	C_max_ (pg/mL)	374 (n = 15)	263 (n = 16)	1.42 (1.24, 1.63)	578 (n = 16)	446 (n = 16)	1.30 (1.13, 1.49)
	AUC_0–24 h_ (h × pg/mL)	1070 (n = 15)	836 (n = 16)	1.28 (1.11, 1.48)	3250 (n = 16)	2680 (n = 16)	1.21 (1.05, 1.40)
Analyte: glycopyrronium							
IND/GLY/MF high-dose	C_max_ (pg/mL)	267 (n = 14)	185 (n = 15)	1.45 (1.16, 1.81)	424 (n = 14)	308 (n = 15)	1.38 (1.13, 1.69)
	AUC_0–24 h_ (h × pg/mL)	270 (n = 14)	263 (n = 15)	1.02 (0.860, 1.22)	739 (n = 14)	702 (n = 15)	1.05 (0.920, 1.20)
IND/GLY/MF medium-dose	C_max_ (pg/mL)	279 (n = 16)	185 (n = 16)	1.51 (1.22, 1.87)	437 (n = 16)	284 (n = 16)	1.54 (1.27, 1.87)
	AUC_0–24 h_ (h × pg/mL)	285 (n = 16)	260 (n = 16)	1.10 (0.925, 1.30)	755 (n = 16)	712 (n = 16)	1.06 (0.929, 1.21)
Analyte: mometasone furoate							
IND/GLY/MF high-dose	C_max_ (pg/mL)	195 (n = 14)	193 (n = 15)	1.01 (0.920, 1.11)	264 (n = 14)	247 (n = 15)	1.07 (0.969, 1.18)
	AUC_0–24 h_ (h × pg/mL)	1700 (n = 14)	1600 (n = 15)	1.07 (0.964, 1.18)	2520 (n = 14)	2180 (n = 15)	1.15 (1.05, 1.27)
IND/GLY/MF medium-dose	C_max_ (pg/mL)	104 (n = 16)	96.3 (n = 16)	1.08 (0.990, 1.18)	139 (n = 16)	121 (n = 16)	1.15 (1.04, 1.26)
	AUC_0–24 h_ (h × pg/mL)	926 (n = 16)	784 (n = 16)	1.18 (1.07, 1.30)	1260 (n = 16)	1030 (n = 16)	1.23 (1.12, 1.34)

Model: log (pk) = ethnic group + sequence + period + treatment + ethnic group * treatment, with random effects for subject and matched pair. Covariates were not included in this analysis. IND/GLY/MF medium-dose, IND/GLY/MF 150/50/80 μg o.d; IND/GLY/MF high-dose, IND/GLY/MF 150/50/160 μg o.d. AUC, area under curve; C_max_, maximum plasma concentration; IND/GLY/MF, indacaterol/glycopyrronium/mometasone. N varies across treatments and ethnicities based on treatment discontinuations during the study as described under study participants. For two Japanese subjects (one profile for the high dose treatment and one profile for the medium dose treatment) for whom pre-dose indacaterol concentration on Day 1 was missing, Day 1 PK parameters were excluded from primary analysis

#### Glycopyrronium

Comparable mean GLY concentration time profiles were observed between treatment groups (IND/GLY/MF medium and high dose) in both Japanese and Caucasian subjects. The mean concentrations over 24 h were slightly higher in Japanese subjects than in Caucasian subjects (Fig. 2b). After single and multiple doses of IND/GLY/MF (either medium or high dose) in Japanese and Caucasian subjects, the median T_max_ of GLY was 0.0833 h both on Day 1 and on Day 14. The accumulation ratios (R_acc_ for AUC and C_max_) after o.d. dosing for 14 days were 2.74–2.86 (AUC_0–24 h_) and 1.69–1.80 (C_max_) in Japanese subjects, and were 2.83–2.84 (AUC_0–24 h_) and 1.68–1.90 (C_max_) in Caucasian subjects (Table 2). The geometric mean ratios (Japanese/Caucasian) of C_max_ on Day 14 in IND/GLY/MF high- and IND/GLY/MF medium-dose treatment groups were 1.38 (90% CI 1.13–1.69) and 1.54 (90% CI 1.27–1.87), respectively. The geometric mean ratios (90% CI) (Japanese/Caucasian) for AUC_0–24 h_ on Day 14 in IND/GLY/MF high- and IND/GLY/MF medium-dose treatment groups were 1.05 (90% CI 0.92–1.20) and 1.06 (90% CI 0.93–1.21), respectively. The geometric mean C_max_ was up to 1.54-fold higher in Japanese than Caucasians subjects, while the geometric mean AUC_0–24 h_ was comparable. A similar trend was observed following a single dose of IND/GLY/MF medium and high dose on Day 1 (Table 3). Based on the results of the exploratory statistical analysis including age and bodyweight as covariates, the geometric mean ratios (90% CI) (Japanese/Caucasian) for C_max_ on Day 14 in IND/GLY/MF medium- and high-dose treatment groups for GLY were 1.33 (90% CI 1.09–1.61) and 1.20 (90% CI 0.98–1.46), respectively. The mean ratios (90% CI) (Japanese/Caucasian) for AUC_0–24 h_ on Day 14 in IND/GLY/MF medium- and high-dose groups were 0.95 (90% CI 0.84–1.09) and 0.95 (90% CI 0.83–1.08), respectively.

### Mometasone furoate

After single and multiple doses of IND/GLY/MF (either medium or high-dose) in Japanese and Caucasian subjects, the median time to reach T_max_ of MF ranged from 1 to 2 h both on Day 1, and on Day 14. There was no difference in T_max_ between Japanese and Caucasian subjects (overall range: 0.25 to 3 h both on Day 1 and Day 14) (Fig. 2c, Table 2). A twofold increase in dose of MF from 80 μg to 160 μg led to an approximately twofold increase in exposure (C_max_ and AUC_0–24 h_) in both Japanese and Caucasian subjects (Fig. 2c). The accumulation ratios (R_acc_ for AUC_0–24 h_ and C_max_) after o.d. dosing of IND/GLY/MF medium dose for 14 days were 1.37 (AUC_0–24 h_) and 1.35 (C_max_) in Japanese subjects, and were 1.33 (AUC_0–24 h_) and 1.28 (C_max_) in Caucasian subjects. The accumulation ratios (R_acc_ for AUC_0–24 h_ and C_max_) after o.d. dosing of IND/GLY/MF high dose for 14 days were 1.50 (AUC_0–24 h_) and 1.38 (C_max_) in Japanese subjects, and were 1.40 (AUC_0–24 h_) and 1.31 (C_max_) in Caucasian subjects (Table 2). The geometric mean ratios (Japanese/Caucasian) for C_max_ on Day 14 in IND/GLY/MF medium- and IND/GLY/MF high-dose treatment groups were 1.15 (90% CI 1.04–1.26) and 1.07 (90% CI 0.97–1.18), respectively. The geometric mean ratios of AUC_0–24 h_ on Day 14 in IND/GLY/MF high- and IND/GLY/MF medium-dose treatment groups were 1.15 (90% CI 1.05–1.27) and 1.23 (90% CI 1.12–1.34), respectively. A similar trend was observed following a single dose of IND/GLY/MF medium dose and IND/GLY/MF high dose on Day 1 (Table 3). The results of the exploratory statistical analysis including age and bodyweight as covariates for C_max_ on Day 14 in IND/GLY/MF medium- and high-dose treatment groups for MF were 1.10 (90% CI 0.99–1.23) and 1.03 (90% CI 0.92–1.15), respectively. The geometric mean ratios (90% CI) (Japanese/Caucasian) for AUC_0–24 h_ on Day 14 in IND/GLY/MF medium and high dose were 1.16 (90%CI, 1.05–1.28) and 1.09 (90% CI 0.99–1.20), respectively.

In Japanese subjects, the geometric mean ratios (high dose [160 μg] versus medium dose [80 μg]) (90%CI) of C_max_ and AUC_0–24 h_ on Day 14 were 1.91 (1.79–2.02) and 2.00 (1.92–2.09), respectively. In Caucasian subjects, the geometric mean ratios (90%CI) for C_max_ and AUC_0–24 h_ on Day 14 were 2.04 (1.93–2.16) and 2.13 (2.04–2.21), respectively (Table 4).

**Table 4:** Summary of the exploratory analysis of plasma MF PK parameters (high dose [160 μg] versus medium dose [80 μg] on Day 1 and Day 14)

Ethnic group	PK parameter (unit)	Day 1			Day 14		
		Adjusted geometric mean	Adjusted geometric mean ratio (test/reference)		Adjusted geometric mean	Adjusted geometric mean ratio (test/reference)	
		IND/GLY/MF high-dose (test)	IND/GLY/MF medium-dose (reference)	Estimate (90% CI)	IND/GLY/MF high-dose (test)	IND/GLY/MF medium-dose (reference)	Estimate (90% CI)
Japanese	C_max_ (pg/mL)	195 (n = 14)	104 (n = 16)	1.87 (1.73, 2.02)	264 (n = 14)	139 (n = 16)	1.91 (1.79, 2.02)
	AUC_0–24 h_ (hxpg/mL)	1700 (n = 14)	926 (n = 16)	1.84 (1.72, 1.97)	2520 (n = 14)	1260 (n = 16)	2.00 (1.92, 2.09)
Caucasian	C_max_ (pg/mL)	193 (n = 15)	96.3 (n = 16)	2.00 (1.86, 2.16)	247 (n = 15)	121 (n = 16)	2.04 (1.93, 2.16)
	AUC_0–24 h_ (hxpg/mL)	1600 (n = 15)	784 (n = 16)	2.03 (1.90, 2.17)	2180 (n = 15)	1030 (n = 16)	2.13 (2.04, 2.21)

Model: log (pk) = ethnic group + sequence + period + treatment + ethnic group * treatment, with random effects for subject and matched pair. Covariates were not included in this analysis. IND/GLY/MF medium-dose, IND/GLY/MF 150/50/80 μg o.d; IND/GLY/MF high-dose, IND/GLY/MF 150/50/160 μg o.d. AUC, area under curve; C_max_, maximum plasma concentration; IND/GLY/MF, indacaterol/glycopyrronium/mometasone furoate

### Safety

IND/GLY/MF was well tolerated and had a similar safety profile at both doses in Japanese and Caucasian subjects. No AEs reported during the study was considered to be related to the study drug by the Investigator and all AEs were resolved by the end of the study. There were no deaths or SAEs reported in the study. One Japanese subject receiving IND/GLY/MF medium-dose reported two AEs (gastroenteritis and headache) that led to discontinuation of study treatment.

## Discussion

We report the results of a Phase I study providing pharmacokinetic data for the LABA/LAMA/ICS combination of IND/GLY/MF in Japanese and Caucasian subjects. This multiple-dose study assessed the PK of IND, GLY, MF on Day 1 and Day 14 following IND/GLY/MF o.d. dosing for 14 days. The study included only healthy male subjects in accordance with local clinical practice and regulatory considerations for Phase I healthy volunteer studies in Japan. Healthy subjects were selected instead of asthma patients to minimize impact of confounding variables (eg: disease conditions, concomitant medications). This was considered the most sensitive approach to investigate the effect of ethnicity on PK of the components of IND/GLY/MF.

### Analysis of individual data across treatments and analytes

Individual concentration–time profiles and individual PK parameters across analytes (IND, GLY or MF) and treatments (150/50/80 μg or 150/50/160 μg) were explored as part of data analysis. There was no correlation between the exposure of the different analytes as evidenced by the lack of any systematic trends for higher exposure for all analytes in an individual subject. These data confirmed generally consistent use of inhalation technique across subjects. Similar accumulation was noted in an individual subject for a particular analyte in both treatments; however, the extent of accumulation was different for different analytes. This observation is consistent with the slightly different mean accumulation ratios for AUC and C_max_ for the 3 analytes (Table 2). The exposure of IND and GLY on Day 1 was comparable within an individual subject across both 150/50/80 μg and 150/50/160 μg treatments, suggesting that the intra-subject variability of IND and GLY exposure was low. Comparable IND and GLY exposure on Day 1 within an individual subject across both 150/50/80 μg and 150/50/160 μg treatments also confirmed that there was no notable period effect and that inhaler device use was consistent across treatment periods.

#### Total systemic exposure (AUC_0–24 h, ss_)

The geometric mean ratios (Japanese/Caucasian) of steady-state AUC_0–24 h_ on Day 14, across both dose groups were in the range of 1.17 to 1.21 for IND, 1.05 to 1.06 for GLY, and 1.15 to 1.23 for MF. Mean bodyweight of Caucasian subjects was slightly higher (approximately 10%) than that of Japanese subjects. This was considered one of the factors that contributed to the slightly higher exposures to all three analytes in Japanese subjects. Based on the results of exploratory statistical analysis including age and bodyweight as covariates, the geometric mean ratios (Japanese/Caucasian) of steady-state AUC_0–24 h_ on Day 14, across both dose groups were in the range of 1.12 to 1.17 for IND, 0.948 to 0.954 for GLY and 1.09 to 1.16 for MF. Overall, the mean total systemic exposure (AUC_0–24 h,ss_) in Japanese healthy subjects increased by 23% at a maximum as compared to Caucasian subjects, across all analytes.

#### Peak systemic exposure (C_max_)

The geometric mean ratios (Japanese/Caucasian) of steady-state C_max_ on Day 14, across both dose groups were in the range of 1.30 to 1.31 for IND, 1.38 to 1.54 for GLY, and 1.07 to 1.15 for MF. Based on the results of exploratory statistical analysis including age and bodyweight as covariates, the geometric mean ratios (Japanese/Caucasian) of steady-state C_max_ on Day 14, across both dose groups were in the range of 1.20 to 1.22 for IND, 1.20 to 1.33 for GLY and 1.03 to 1.16 for MF. Interpretation of C_max_ data is challenging for inhaled drugs with rapid absorption in the lungs. For example, GLY reaches peak plasma levels at 5 min post-dose, at the first sampling point post-inhalation. This reflects the instantaneous absorption of GLY in the lungs. Due to the rapid absorption, small differences in the actual sampling time can have a large impact on the apparent C_max_. The planned assessment window for the 5 min post-dose sampling time point was ± 3 min. While there were no deviations from the sampling time noted during the study, a narrower assessment window would have potentially led to lesser variability in MF C_max_ than that observed in the current study (CV% range 28.9–53.5%, Table 2).

The results of this study are consistent with the finding for IND and MF exposure reported in a previous ethnic sensitivity study where the fixed-dose combination of IND and MF (QMF149 150/80 μg or 150/320 μg) administered via the Breezhaler^®^ device was evaluated in Japanese and Caucasian subjects [23]. IND and MF are primarily metabolized via the CYP3A4 [24] whereas GLY is predominantly metabolized by CYP2D6. No ethnic variation has been reported for the expression and polymorphisms of CYP3A4, the observed difference in the IND and MF exposure was not considered to be due to any ethnic differences in a metabolic process.

Single and multiple doses of IND/GLY/MF medium and high dose were safe and well tolerated in Japanese and Caucasian healthy subjects. Drug-related AEs or SAEs were not reported in the study.

Overall, no clinically relevant ethnic effects on the systemic exposure to IND, GLY and MF was observed after multiple-dose administration of IND/GLY/MF in healthy subjects. The observed differences were small in magnitude and were not considered clinically relevant in view of the observed exposure variability, challenges with characterizing C_max_ for inhaled drugs and the available safety data at doses higher than proposed doses of IND [25], GLY [26] and MF [27] as part of IND/GLY/MF. In the Phase 3 clinical study for IND/GLY/MF in asthma patients [28], while the study was not powered to demonstrate differences by subgroups, there were no observed differences in clinical efficacy or safety profile according to recorded ethnicity. In addition, during the development of IND/GLY/MF in Japan, 52 weeks treatment of IND/GLY/MF was well tolerated in Japanese patients with inadequately controlled asthma [29]. Overall adverse event profile was generally consistent with that known to occur with one or more of the components. No new or unexpected safety signals were observed in the study. Furthermore, a literature search to assess the potential risk of safety concerns in Japanese subjects resulting from an increased exposure for IND, GLY or MF did not reveal any safety concerns specific to the Japanese population following single or multiple dose administration of IND [18, 30], GLY [31] or the IND/GLY combination [32, 33] in COPD patients. The observed adverse events were expected in the patient population studied and were similar to those seen in studies conducted in the Caucasian population. Similarly, there were no safety concerns specific to the Japanese population in a single dose study with IND [34] or a multiple dose study with MF in Japanese asthma patients [35]. Overall, based on the literature evidence, the safety profile for IND, GLY and MF in monotherapy or combination products was similar between Japanese and Caucasian populations. No potential risk of safety concerns was identified in Japanese subjects due to increased exposure of IND, GLY or MF.

This study was conducted as a parallel group evaluation in Japanese and Caucasian subjects in a single center with demographically matched subjects from both ethnic groups. Assessment of PK in both ethnic groups at the same site within the same study avoids inter-study or inter-center variability, which may contribute to an apparent difference in PK of inhaled compounds due to potential site-specific differences in inhalation technique used by study volunteers. In prior studies for MF delivered with the Twisthaler^®^ inhalation device, the C_max_ and AUC values were about two to threefold higher for Japanese patients with asthma compared with Caucasian patients with asthma [20]. This difference in approach to study conduct (single center vs. cross-study comparisons based on separate studies in Caucasian and Japanese patients) may explain the differences noted between prior data and our analysis we noted no clinically relevant difference between MF AUC and C_max_ between ethnicities.

The mean total and peak exposures of IND and GLY observed in this study were higher than noted in previous studies [38]. Mean C_max_ and AUC_0–24 h_ of GLY on Day 14 following administration of GLY alone in asthma patients (Caucasian) were 166 pg/mL and 464 h × pg/mL, respectively. Mean C_max_ and AUC_0–24 h_ of IND on Day 14 following administration of IND alone or as part of the IND/MF fixed-dose combination were 307–317 pg/mL and 1910–1950 h × pg/mL, respectively, in healthy subjects. However, acknowledging limitations of cross-study comparisons, overall the observed exposure levels are consistent with those observed in previous PK studies.

## Conclusions

IND, GLY and MF were systemically available shortly after the inhalation of single or multiple doses of IND/GLY/MF at the medium and high ICS dose strengths in both Japanese and Caucasian subjects. Median plasma T_max_ for IND, GLY and MF across both dose groups was 15 min, 5 min and 1–2 h, respectively. Slightly higher exposure of each individual drug in Japanese subjects as compared with Caucasian subjects was observed following single or multiple doses of IND/GLY/MF medium or high dose but is not considered clinically relevant. For MF, C_max_ and AUC_0–24 h_ increased in a dose-proportional manner between IND/GLY/MF medium and high dose on both Day 1 and Day 14 for both ethnicities. Single and multiple doses of IND/GLY/MF medium and high dose were safe and well tolerated in Japanese and Caucasian healthy subjects.

## Supplementary Information

**Additional file 1.** Analytical parameters and performances of the three methods used for IND, GLY and MF.

## Data Availability

Novartis is committed to sharing with qualified external researchers, access to patient-level data and supporting clinical documents from eligible studies. These requests are reviewed and approved by an independent review panel on the basis of scientific merit. All data provided are anonymized to respect the privacy of subjects who have participated in the trial in line with applicable laws and regulations. Result summaries have been posted on the Novartis clinical trial database and other online public databases. More information on Novartis’ position on access to clinical trial results and patient-level data is available at: https://www.novartis.com/our-science/clinical-trials/clinical-trial-information-disclosure

## Abbreviations

AE: Adverse event
AUC: Area under the curve
BMI: Body Mass Index
COPD: Chronic obstructive pulmonary disease
C_max_: The observed maximum plasma concentration following drug administration
ECG: Electrocardiogram
FPM: Fine particle mass
GINA: Global INitiative for Asthma
GLY: Glycopyrronium bromide
ICS: Inhaled corticosteroid
IND: Indacaterol acetate
LABA: Long-acting β agonist
LAMA: Long-acting muscarinic antagonist
LTRA: Leukotriene receptor antagonists
LLOQ: Lower limit of quantification
MF: Mometasone furoate
o.d.: Once-daily
PK: Pharmacokinetics
R_acc_: The accumulation ratio
SAE: Serious adverse event
T_max_: The time to reach the maximum concentration after drug administration
