# Supplementary material for: Pharmacokinetics of indacaterol, glycopyrronium and mometasone furoate administered as an inhaled fixed-dose combination in Japanese and Caucasian healthy subjects
Source: BMC Pulm Med. 2021 Jan 7;21:18. doi: 10.1186/s12890-020-01382-6 (PMC7791651; doi:10.1186/s12890-020-01382-6)
Supplement: Supplementary file 1 — Additional file 1. Analytical parameters and performances of the three methods used for IND, GLY and MF. [file 12890_2020_1382_MOESM1_ESM.docx]

**Supplementary Appendix**

**Supplementary Table 1**. **Analytical parameters and performances of the three methods used for IND, GLY and MF**

| **Compound** | **Analytical conditions -** | **Calibration range** | **MS Parameters** | **Bias (%)^a^** | **Precision (%)^b^** |
| --- | --- | --- | --- | --- | --- |
| Indacaterol | Sample processing solid phase extraction on Oasis Mixed mode (10 mg, 30 µm) cartridge using a sample volume of 200 µL.  Separation: Acquity UPLC^®^ BEH C_18_ 1.7µM at 40°C and using 0.1% formic acid in water as mobile phase A, acetonitrile as mobile phase B and operating at a gradient with an initial flow rate of 0.750 mL/min.  A triple quadrupole mass spectrometer (API 6500) equipped with a turbo ion spray source is used for detection in positive ion mode.  Quantification is based on multiple reaction monitoring (MRM) of the transitions of m/z 392.9 – 173.2 | 5.00 to 5000 pg/mL  A linear calibration curve with a 1/x^2^ weighting | Curtain gas 50 units  GS1 (nebulizing gas, N_2_) 50 units;  GS2 (desolvation gas, N_2_) 65 units  Ion spray voltage 5000 V;  Source Temperature: 500°C  Declustering  Potential 50 V  Collision  Energy 30 V  Collision exit  Potential 20 V | 1.9 – 2.4 | 0.3 – 2.4 |
| Glycopyrronium | Sample processing: solid phase extraction on Oasis weak cationic ion exchange (10 mg, 30 µm) cartridge using a sample volume of 200 µL.  Separation: ACQUITY UPLC BEH C_18_ analytical column at 45°C and using 0.1% formic acid in water as (mobile phase A) and acetonitrile as (mobile phase B) operating at a gradient with a flow rate of 0.750 mL/min. Total run time 3 min.  Instrument: triple quadrupole 6500 mass spectrometer equipped with a turbo ion spray source used for detection in positive mode  Quantification: multiple reaction monitoring (MRM) of the transitions of m/z 318.0 – 116.1. | 1.00 to 1000 pg/mL  A linear calibration curve with a 1/x^2^ weighting | Curtain gas 35 units;  GS1 (nebulizing gas, N_2_) 50 units;  GS2 (desolvation gas, N_2_) 65 units;  Ion spray voltage 5000 V;  Source Temperature: 500°C  Declustering  Potential 100 V  Collision  Energy 35 V  Collision exit  Potential 20 V | -4.8 – 4.7 | 1.7 – 3.7 |
| Mometasone furoate | Sample processing: liquid-liquid extraction using a sample volume of 800 µL plasma  Separation: Acquity UPLC BEH C_18_ column at 60°C, using 0.05% ammonia in water as (mobile phase A), and acetonitrile (mobile phase B), operating at a gradient with a flow rate of 1.00 mL/min. Total run time 3.5 min.  Instrument: triple quaduprole 6500 mass spectrometer equipped with a turbo ion spray source is used for detection in positive ion mode. Quantification: MRM using transitions of m/z 521.1 → 355.2 | 0.250 - 100 pg/mL  A linear calibration curve with a 1/x^2^ weighting | Curtain gas 25 units;  GS1 (nebulizing gas, N_2_) 65 units;  GS2 (desolvation gas, N_2_) 60 units;  Ion spray voltage 3500 kV;  Declustering  Potential 30 V  Collision  Energy 21 V  Collision exit  Potential 10 V | 3.1 – 4.0 | 0.5 – 5.9 |

^a^Data obtained during the three validation runs

^b^Data obtained during the three validation runs
